# Controlled ovarian hyperstimulation parameters are not associated with *de novo* chromosomal abnormality rates and clinical pregnancy outcomes in preimplantation genetic testing

**DOI:** 10.3389/fendo.2022.1080843

**Published:** 2023-01-12

**Authors:** Yanli Liu, Junhan Shen, Yuchao Zhang, Rui Peng, Junliang Zhao, Pengfei Zhou, Rujing Yang, Yichun Guan

**Affiliations:** ^1^ The Reproduction Center, The Third Affiliated Hospital of Zhengzhou University, Zhengzhou, China; ^2^ Office of Scientific Research, The Third Affiliated Hospital of Zhengzhou University, Zhengzhou, China

**Keywords:** controlled ovarian hyperstimulation (COH), *in vitro* fertilization, preimplantation genetic testing (PGT), *de novo* chromosomal abnormality, clinical pregnancy outcomes

## Abstract

**Objective:**

This study aimed to determine whether controlled ovarian hyperstimulation (COH) parameters influence the incidence of *de novo* chromosomal abnormalities (> 4 Mb) in blastocysts and, thus, clinical pregnancy outcomes in preimplantation genetic testing (PGT).

**Methods:**

Couples who underwent preimplantation genetic testing for structural chromosome rearrangements (PGT-SR) and monogenic disorders (PGT-M) were included in this study. The relationships of maternal age, paternal age, stimulation protocol, exogenous gonadotropin dosage, duration of stimulation, number of oocytes retrieved and estradiol (E_2_) levels on human chorionic gonadotropin (hCG) trigger day with the incidence of *de novo* chromosomal abnormalities were assessed. Blastocysts were biopsied, and nuclear DNA was sequenced using next-generation sequencing (NGS). Clinical pregnancy outcomes after single euploid blastocyst transfers under different COH parameters were assessed.

**Results:**

A total of 1,710 and 190 blastocysts were biopsied for PGT-SR and PGT-M, respectively. The rate of *de novo* chromosomal abnormalities was found to increase with maternal age (*p<* 0.001) and paternal age (*p* = 0.019) in the PGT-SR group. No significant differences in the incidence of *de novo* chromosomal abnormalities were seen for different maternal or paternal age groups between the PGT-SR and PGT-M groups (*p* > 0.05). Stratification analysis by gonadotropin dosage, stimulation protocol, duration of stimulation, number of retrieved oocytes and E_2_ levels on hCG trigger day revealed that *de novo* chromosomal abnormalities and clinical pregnancy outcomes were not correlated with COH parameters after adjusting for various confounding factors.

**Conclusion:**

The rate of *de novo* chromosomal abnormalities was found to increase with maternal or paternal age. COH parameters were found to not influence the incidence of *de novo* chromosomal abnormalities or clinical pregnancy outcomes.

## Introduction

In assisted reproductive technology (ART), aneuploidy is one of the most significant causes of pregnancy failure and miscarriage. Aneuploidy occurs due to meiotic errors during gametogenesis, the fertilization of unbalanced gametes or mitotic errors during embryonic development (1, 2). Therefore, a major goal of controlled ovulation hyperstimulation (COH) is to achieve maximal follicular development during a single menstrual cycle. It is thought that aneuploidy can be avoided through the collection of many oocytes, thereby increasing the likelihood of obtaining euploid embryos. However, embryonic chromosomal abnormalities are thought to also occur due to iatrogenic factors (3), with the *in vitro* fertilization (IVF) process itself and associated COH increasing the risk of meiotic or mitotic errors. It has been speculated that the use of exogenous gonadotropins (Gn) to stimulate multifollicular development can interfere with the natural selection of dominant follicles, thereby increasing the retention of aneuploid oocytes (4, 5). Moreover, COH has been hypothesized to increase oocyte division errors and affect genomic imprinting (6). However, the specific nature of such adverse effects of COH remains unclear.

As preimplantation genetic testing (PGT) is an important prenatal diagnostic method used to detect aneuploid embryos (7), it can prevent the transmission of pathogenic genetic mutations or an unbalanced chromosome to the offspring, increasing the chances of a successful, healthy pregnancy. Therefore, PGT is widely used in clinical practice (7, 8). Many studies have explored the relationship between COH and aneuploid, but have yielded inconclusive results. It has been suggested that higher Gn dosage increases the incidence of aneuploid (9, 10). In contrast, other studies have reported that the incidence of embryonic aneuploidy was not affected by exogenous Gn, with equivalent aneuploidy rates across unstimulated and stimulated IVF cycles, and different Gn dosages (11, 12). However, most studies have focused on the relationship between ovulation induction and aneuploidy (6, 13), although many other intrinsic factors can influence ploidy. Aneuploidy can be inherited from a parental carrier of genetic abnormalities (14), or it can occur *de novo (* 15, 16). PGT for aneuploidy (PGT-A) is typically carried out following recurrent miscarriages and repeated implantation failures or in cases of advanced maternal age. In contrast, PGT for structural chromosome rearrangements (PGT-SR) is carried out for individuals known to have high rates of unbalanced gametes after meiotic segregation, resulting in embryos with abnormal chromosomal composition. Therefore, we sought to study the effect of COH on the rate of *de novo* chromosomal abnormalities identified by PGT-SR. As patients undergoing PGT for monogenic disorders (PGT-M) have known monogenetic mutations but normal karyotypes, they typically do not have fertility issues and are not at risk of elevated embryonic aneuploidy rates. We therefore used patients undergoing PGT-M as our control group.

In this study, we aimed to determine whether COH parameters are associated with *de novo* chromosomal abnormality rates in the Chinese population served by our reproductive center at The Third Affiliated Hospital of Zhengzhou University. We compared patients undergoing unstimulated and stimulated PGT-SR and PGT-M and explored various COH parameters for their associations with aneuploidy rates and clinical pregnancy outcomes. To our knowledge, this is the first systematic evaluation of the effects of specific COH parameters on *de novo* chromosomal abnormalities rates and clinical pregnancy outcomes involving PGT using next-generation sequencing (NGS) techniques. This research will inform the development of improved stimulation protocols that may reduce the occurrence of *de novo* aneuploidy following COH.

## Materials and methods

### Study population

This retrospective study involved 430 patients who underwent PGT-SR and 47 patients who underwent PGT-M from January 2017 to December 2021 (**Table 1**). Anonymous data were obtained from the Center for Reproductive Medicine at The Third Affiliated Hospital of Zhengzhou University. Chromosomal karyotyping of all participants was performed by standard Giemsa banding techniques prior to ovarian stimulation. Couples where at least one partner was a known carrier of a reciprocal translocation (REC), Robertsonian translocation (ROB) or inversion (INV) were assigned to the PGT-SR group. Patients with monogenetic mutations but normal karyotypes were assigned to the PGT-M group. Basic patient information was gathered, and other parameters, including the number of oocytes retrieved, fertilization rate, embryo formation rate and blastocyst formation rate, were recorded.

**Table 1:** Patient characteristics and corresponding PGT data.

	PGT-SR	PGT-M	t/χ^2^	*P*
No. of cycles	430	47		
Maternal age (years, $\bar{x}$ ± SD)	30.94 ± 4.06	30.83 ± 3.78	0.373	0.709
Maternal BMI (years, $\bar{x}$ ± SD)	23.56 ± 3.11	23.53 ± 3.15	0.142	0.887
Paternal age (years, $\bar{x}$ ± SD)	31.56 ± 4.01	31.86 ± 3.44	1.109	0.269
Paternal BMI ( $\bar{x}$ ± SD)	25.45 ± 4.08	25.77 ± 4.09	0.970	0.332
FSH (mIU/mL, $\bar{x}$ ± SD)	6.55 ± 1.71	6.72 ± 1.51	1.292	0.196
LH (mIU/mL, $\bar{x}$ ± SD)	6.28 ± 4.06	6.66 ± 2.85	1.671	0.096
E_2_ (pmol/L, $\bar{x}$ ± SD)	147.44 ± 86.69	139.35 ± 57.12	1.256	0.209
AMH (pmol/L, $\bar{x}$ ± SD)	28.57 ± 14.24	27.20 ± 13.51	1.312	0.191
No. of retrieved oocytes (n)	6,899	733		
No. of blastocysts for PGT analysis (n)	1,734	191		
Oocytes in MII stage (%, n)	79.58% (5,490/6,899)	79.26% (581/733)	0.040	0.841
2PN fertilized oocytes (%, n)	81.77% (4,489/5,490)	81.58% (474/581)	0.012	0.913
Day 3 available embryos (%, n)	80.98% (3,635/4,489)	79.54% (377/474)	0.574	0.449
Available blastocysts (%, n)	49.63% (1,804/3,635)	61.01% (230/377)	17.696	<0.001*
Blastocysts with genetic results (%, n)	98.62% (1,710/1,734)	99.48% (190/191)	0.994	0.319
Euploid blastocysts (%, n)	33.63% (575/1,710)	60.53% (115/190)	53.505	<0.001*
Aneuploid blastocysts (%, n)	55.03% (941/1,710)	23.68% (45/190)	67.300	<0.001*
Mosaic blastocysts (%, n)	11.34% (194/1,710)	15.79% (30/190)	3.248	0.072
No. of blastocysts with *de novo* chromosomal abnormalities (%, n)	22.98% (393/1,710)	22.63% (43/190)	0.012	0.913

Value are presented as means ± standard deviations or number, n (%).

*p< 0.05 was considered statistically significant with respect to the PGT-SR group.

PGT-SR, Preimplantation Genetic Testing for Structural Rearrangements; PGT-M, Preimplantation Genetic Testing for Monogenic; BMI, body mass index; FSH, follicle-stimulating hormone; LH, luteinizing hormone; E_2_, estradiol; AMH, Anti-Mullerian hormone; PGT, Preimplantation Genetic Testing; MII, second metaphase; PN, pronucleus.

### Ethics approval

The study was reviewed and approved by the Ethics Committee of the Third Affiliated Hospital of Zhengzhou University, and all patients underwent genetic counseling. Written informed consent for participation was not required for this study, in accordance with national legislation and institutional requirements.

### Basic clinical characteristics

Baseline demographic information was collected, including maternal and paternal age (years, y) and maternal and paternal body mass index (BMI; kg/m^2^). Basal plasma follicle stimulating hormone (FSH; mIU/mL), luteinizing hormone (LH; mIU/mL) and estradiol (E_2_; pmol/L) concentrations were measured on day two or three of the menstrual cycle, and anti-Mullerian hormone (AMH; pmol/L) concentrations were measured on any day of the menstrual cycle. COH parameters, including the ovulation stimulation protocol used, Gn dosage (IU), duration of ovarian stimulation (days, d), number of retrieved oocytes and peak E_2_ concentrations (pmol/L), were documented for all patients.

### Controlled ovarian hyperstimulation

Ovarian stimulation and gonadotrophin (Gn) administration was performed by experienced clinicians taking into consideration maternal age, antral follicle count (AFC), basal FSH concentration, the cause of infertility and ovarian reserve function. COH cycles for PGT require the administration of more Gn to ensure that more oocytes are collected and enough transferable embryos remain after testing. Three stimulation protocols were used: a gonadotropin-releasing hormone (GnRH) antagonist, a GnRH agonist and progestin-primed ovarian stimulation (PPOS). Gn dosing and induction duration were adjusted with ovarian response, as monitored using transvaginal ultrasound and circulating E_2_. Patients were categorized into different groups according to the total amount of Gn administered (< 2,000, 2,000–3,000 and > 3,000 IU), stimulation duration (< 10, 10–12 and > 12 d), number of oocytes retrieved (< 10, 10–15 and > 15 oocytes) and peak E_2_ concentrations (< 10,000, 10,000–15,000 and > 15,000 pmol/L). hCG was administered by injection to promote oocyte maturation when the diameter of at least two follicles exceeded 18 mm.

### Oocyte collection, intracytoplasmic sperm injection (ICSI), embryo culture, and blastocyst biopsy

Transvaginal ultrasonography-assisted oocyte aspiration was performed approximately 36 hours after the hCG injection. After oocyte retrieval, cumulus-oocyte complexes were cultured for 4 h and then inseminated by intracytoplasmic sperm injection (ICSI). If the oocytes were at the metaphase-I (MI) or germinal vesicle (GV) stage, they were cultured *in vitro* until mature for an additional 24 h and then fertilized. Fertilization was confirmed by the presence of two pronuclei (2PN) 17–18 h post-insemination. Embryo cleavage was evaluated 41–44 h (Day 2) and 65–68 h (Day 3) after ICSI. For the first 3 days post-ICSI, the embryos were cultured in G1™ plus (Vitrolife, Sweden) in a humidified incubator with 5% O_2_ and 6% CO_2_. All cleavage embryos were transferred into G2™ plus (Vitrolife, Sweden) sequential media and cultured until they reached the blastocyst stage. Blastocysts were scored using the Gardner grading system. Blastocysts graded above 3BC were used for subsequent biopsies. Trophectoderm biopsies were performed from day 5 to 7 of development, based on the time of gastrulation, using the laser method. For genetic analysis, 5–8 cells were biopsied and analyzed using NGS (17).

### Sample preparation and NGS analysis

The biopsied samples were washed with G-MOPS™ plus medium and placed in 0.2-mL polymerase chain reaction (PCR) tubes with 2 μL PBS. NGS allows direct quantification of the sequenced DNA fragments based on read numbers. In accordance with the Illumina NGS protocol, raw data were further processed using computational bioinformatic algorithms to map and align the short sequence reads to a linear human reference genome sequence. A small minority of cases, for which DNA amplification failed, were excluded from the study. The minimum detection range was 4 Mb. An embryo was considered “abnormal” when the result deviated from the reference baseline. Embryos with< 20%, 20%–80% and > 80% aneuploid cells were classified as euploid, mosaic and aneuploid, respectively. In the case of aneuploid embryos, if the chromosome involved was the same as that of an affected parent, it was classified as a genetic abnormality. In contrast, if the imbalanced chromosome was normal in the parents, it was classified as a *de novo* abnormality.

### Endometrium preparation and frozen embryo transfer

Natural cycle tracking, hormone replacement therapy and ovarian stimulation were used for endometrium preparation. Luteal-phase support was initiated when endometrial thickness reached at least 7 mm and was continued until 3 months of gestation. A single euploid embryo was chosen for transfer. Biochemical pregnancy was defined as a serum concentration of ß-hCG > 30 mIU/ml measured 2 weeks post-embryo transfer. Clinical pregnancy was confirmed by the ultrasonographic observation of a gestational sac 35 days post-embryo transfer. Spontaneous abortion was defined as a pregnancy with a gestational sac that did not result in a live birth. Live birth was defined as the delivery of at least one live birth at ≥ 28 weeks of gestation.

### Statistical analysis

Statistical analyses were performed using SPSS 24.0. Continuous variables are presented as the mean ± standard deviation (SD). Categorical variables are presented as absolute values and percentage frequencies.

The chi-square test was used to compare differences in categorical variables, and one-way analysis of variance (ANOVA) to compare differences in continuous variables, between the groups. *P<* 0.05 was considered statistically significant. The relationships between various COH parameters and the incidence of *de novo* chromosomal abnormalities and clinical pregnancy outcomes were analyzed using logistic regression. ^a^ *p*-values were calculated using a mixed logistic model adjusted for maternal age, maternal BMI and blastocyst quality. ^b^ *p*-values were calculated using a mixed logistic model adjusted for maternal age, maternal BMI, method of endometrial preparation, endometrial thickness transfer day of blastocyst and blastocyst quality.

## Results

### General characteristics of study subjects

From January 2017 to December 2021, 477 PGT cycles were initiated at the study center for which blastocysts were subsequently biopsied. No significant differences were seen between the PGT-M and PGT-SR groups in terms of maternal age or BMI, paternal age or BMI, basal FSH, E_2_ and LH or AMH. A total of 7,632 oocytes were collected, and 6,071 MII oocytes were subsequently used for ICSI. In the PGT-SR and PGT-M groups, 81.77% and 81.58% of MII oocytes were successfully fertilized and developed into normally fertilized oocytes with two pronuclei (2PN), of which 49.63% and 61.01% fertilized oocytes developed into blastocysts suitable for biopsy, respectively. In the PGT-SR group, 1,710 (98.62%) of blastocysts had genetic results, of which 575 (33.63%) were euploid, 941 (55.03%) were aneuploid, 194 (11.34%) were mosaic and 393 (22.98%) had *de novo* chromosomal abnormalities. In the PGT-M group, 190 blastocysts had genetic results, of which 115 (60.53%) were euploid, 45 (23.68%) were aneuploid, 30 (15.79%) were mosaic and 43 (23.63%) had *de novo* chromosomal abnormalities. The total number of blastocysts, number of euploid blastocysts and number of aneuploid blastocysts were significantly different in the PGT-SR and PGT-M groups (all *p<* 0.05), as shown in **Table 1**.

### Parent’s age and the occurrence of *de novo* chromosomal abnormalities

As shown in **Table 2**, no significant differences were seen between the PGT-SR and PGT-M groups in the incidence of *de novo* chromosomal abnormalities in each maternal age group and paternal age group. However, within the PGT-SR group, there was a statistically significant increase in such abnormalities with increasing maternal age (*p*< 0.001). Similarly, the rate of *de novo* chromosomal abnormalities was found to increase with paternal age in the PGT-SR group (p = 0.019). In contrast, in the PGT-M group, the rate of blastocysts with *de novo* chromosomal abnormalities increased with maternal or paternal age, but these differences were not statistically significant (*p* > 0.05).

**Table 2:** Incidence of *de novo* chromosomal abnormality in different maternal and paternal age groups.

	The incidence of *de novo* chromosomal abnormalities			
	PGT-SR cycles	PGT-M cycles	χ^2^	*p*
Maternal age				
<30	17.55% (109/621)	16.39% (10/61)	0.052	0.820
30–35	24.29% (221/910)	24.35% (28/115)	0.000	0.988
>35	35.20% (63/179)	35.71% (5/14)	0.002	0.969
χ2	26.302	2.878		
*p*	<0.001*	0.237		
Paternal age				
<30	20.19% (105/520)	19.61% (10/51)	0.010	0.921
30–35	22.86% (216/945)	23.01% (26/113)	0.001	0.971
>35	29.39% (72/245)	26.92% (7/26)	0.069	0.793
χ2	7.974	0.549		
*p*	0.019*	0.760		

Values are presented as number, n (%).

*p< 0.05 was considered statistically significant.

PGT-SR, Preimplantation Genetic Testing for Structural Rearrangements; PGT-M, Preimplantation Genetic Testing for Monogenic.

### COH parameters and the incidence of *de novo* chromosomal abnormalities

Logistic regression analysis was used to analyze the association between ovarian stimulation factors and the incidence of *de novo* chromosomal abnormalities by adjusting for maternal age and BMI and blastocyst quality (**Table 3**). No significant differences were identified in different stimulation protocols, Gn doses, stimulation durations, number of retrieved oocytes or maximal E_2_ concentrations on hCG trigger day between the PGT-SR and PGT-M groups after adjusting for confounding factors.

**Table 3:** Incidence of *de novo* chromosomal abnormalities for different ovarian hyperstimulation parameters.

	*De novo* chromosomal abnormalities in the PGT-SR group					*De novo* chromosomal abnormalities in the PGT-M group				
	Proportion	*p*	OR (95% CI)	*^a^p*	^a^OR (95% CI)	Proportion	*p*	OR (95% CI)	*^a^p*	^a^OR (95% CI)
Stimulation protocol										
GnRH agonist	22.70% (183/806)	Ref		Ref		18.64% (11/59)	Ref		Ref	
GnRH antagonist	22.94% (167/728)	0.913	1.013 (0.798–1.287)	0.287	1.143 (0.893–1.463)	27.72% (28/101)	0.199	1.674 (0.762–3.676)	0.076	2.127 (0.923–4.900)
PPOS	24.43% (43/176)	0.622	1.101 (0.752–1.612)	0.962	1.010 (0.680–1.498)	13.33% (4/30)	0.529	0.671 (0.194–2.320)	0.924	0.99 (0.257–3.434)
Gn dose (IU)										
<2,000	21.52% (88/409)	Ref		Ref		13.79% (4/29)	Ref		Ref	
2,000-3,000	22.96% (180/784)	0.571	1.087 (0.815–1.451)	0.936	0.988 (0.734–1.329)	23.00% (23/100)	0.289	1.867 (0.589–5.918)	0.478	1.593 (0.440–5.759)
>3,000	24.18% (125/517)	0.339	1.163 (0.853–1.586)	0.397	0.866 (0.620–1.208)	26.23% (16/61)	0.192	2.222 (0.669–7.376)	0.409	1.826 (0.438–7.624)
Stimulation duration (d)										
<10	22.53% (105/466)	Ref		Ref		22.81% (13/57)	Ref		Ref	
10–12	23.82% (187/785)	0.602	1.075 (0.819–1.412)	0.934	0.988 (0.748–1.306)	23.85% (26/109)	0.880	1.060 (0.496–2.266)	0.698	0.851 (0.377–1.923)
>12	22.00% (101/459)	0.847	0.970 (0.712–1.322)	0.203	0.813 (0.592–1.118)	16.67% (4/24)	0.537	0.677 (0.196–2.337)	0.286	0.484 (0.128–1.836)
No. of retrieved oocytes										
<10	29.57% (55/186)	Ref		Ref		21.62% (8/37)	Ref		Ref	
10–15	24.24% (135/557)	0.150	0.762 (0.526–1.103)	0.230	0.792 (0.541–1.160)	22.86% (8/35)	0.900	1.074 (0.353–3.264)	0.834	1.134 (0.350–3.669)
>15	20.99% (203/967)	0.011	0.633 (0.446–0.899)	0.188	0.780 (0.539–1.129)	22.88% (27/118)	0.873	1.076 (0.440–2.627)	0.305	1.765 (0.596–5.228)
Peak E_2_ levels(pmol/L)										
<10,000	24.28% (201/828)	Ref		Ref		25.71% (27/105)	Ref		Ref	
10,000–15,000	20.86% (87/417)	0.178	0.822 (0.619–0.193)	0.483	0.901 (0.674–1.205)	18.18% (6/33)	0.379	0.642 (0.239–1.722)	0.789	0.860 (0.284–2.605)
>15,000	22.58% (105/465)	0.492	0.910 (0.695–1.191)	0.931	1.012 (0.768–1.334)	19.23% (10/52)	0.369	0.688 (0.304–1.557)	0.690	0.837 (0.350–2.006)

Values are presented as number, n (%). Unless otherwise stated, p-values were calculated using a univariable mixed logistic model. ^a^p-values were calculated using a mixed logistic model adjusted for maternal age and BMI and blastocyst quality. ^a^Odds ratios (OR) were adjusted by maternal age and BMI and blastocyst quality. 95% confidence intervals (CI) are provided.

PGT-SR, Preimplantation Genetic Testing for Structural Rearrangements; PGT-M, Preimplantation Genetic Testing for Monogenic; Ref, Reference group; OR, odds ratios; GnRH, gonadotropin-releasing hormone; PPOS, progestin-primed ovarian stimulation; Gn, gonadotropins; E2, estradiol.

### COH parameters and clinical pregnancy outcomes

**Figure 1:**
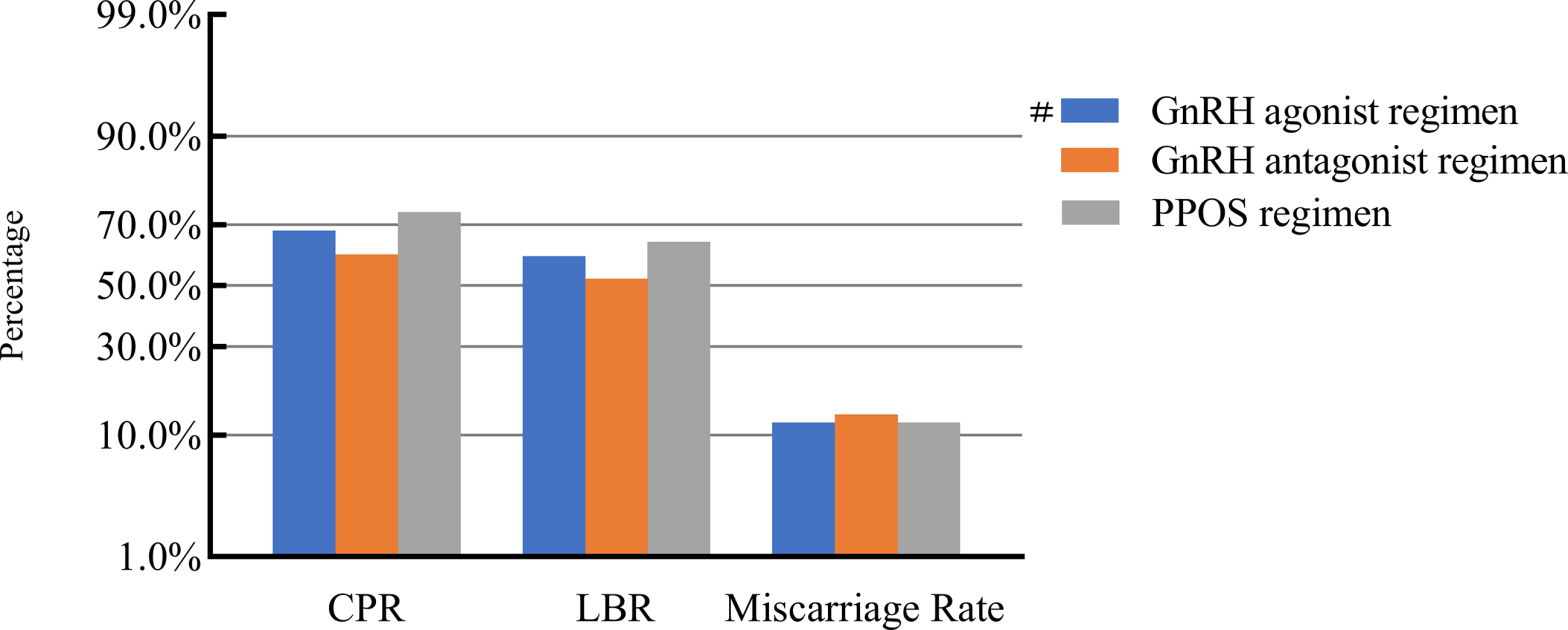
Association between stimulation protocols and pregnancy outcomes. #, Reference group ^b^ *p*-values were calculated using a mixed logistic model adjusted for maternal age and BMI, method of endometrial preparation, endometrial thickness, transfer day of blastocyst and blastocyst quality. CPR, clinical pregnancy rate; LBR, live birth rate; GnRH, gonadotropin-releasing hormone; PPOS, progestin-primed ovarian stimulation.

**Figure 2:**
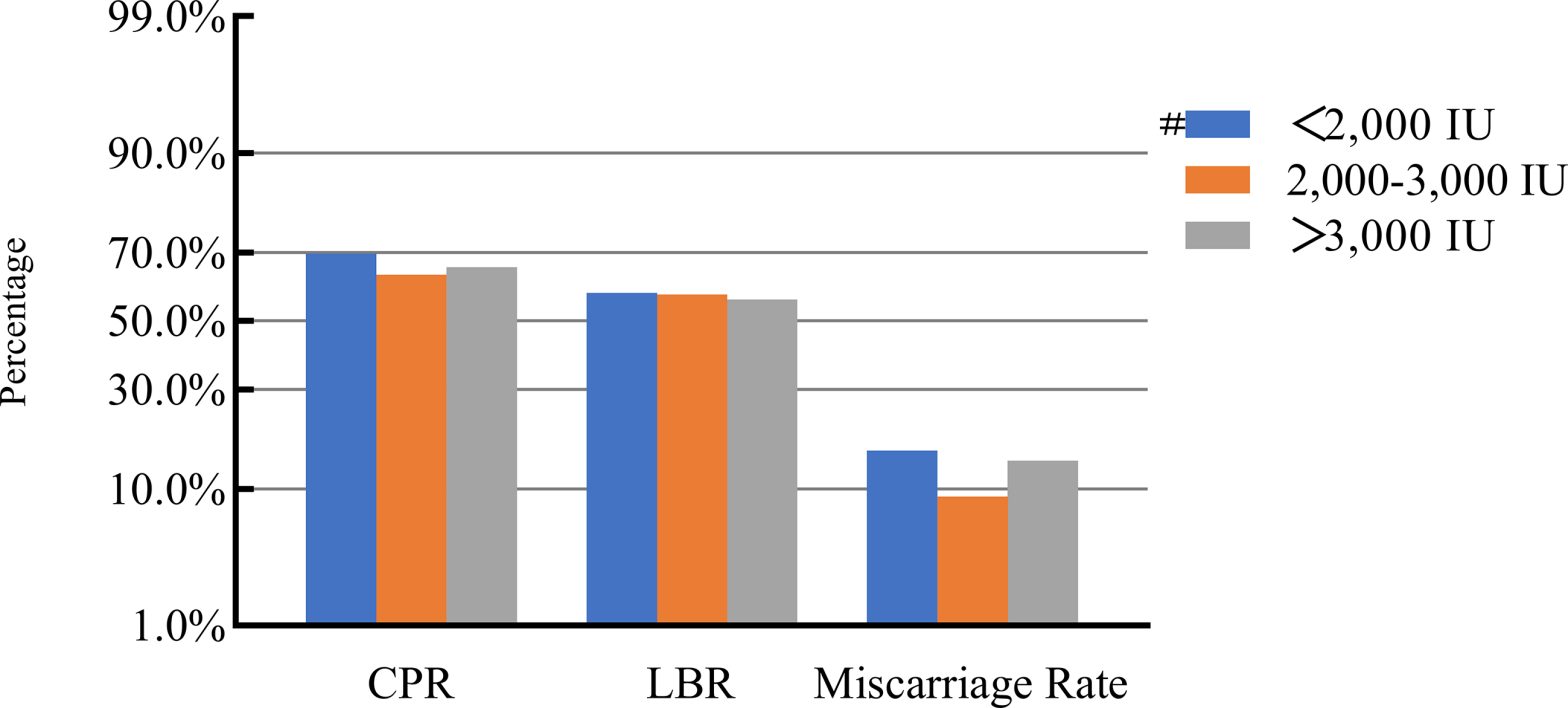
Association between gonadotropin dosage and pregnancy outcomes. #, Reference group ^b^ *p*-values were calculated using a mixed logistic model adjusted for maternal age and BMI, method of endometrial preparation, endometrial thickness, transfer day of blastocyst and blastocyst quality. CPR, clinical pregnancy rate; LBR, live birth rate.

**Figure 3:**
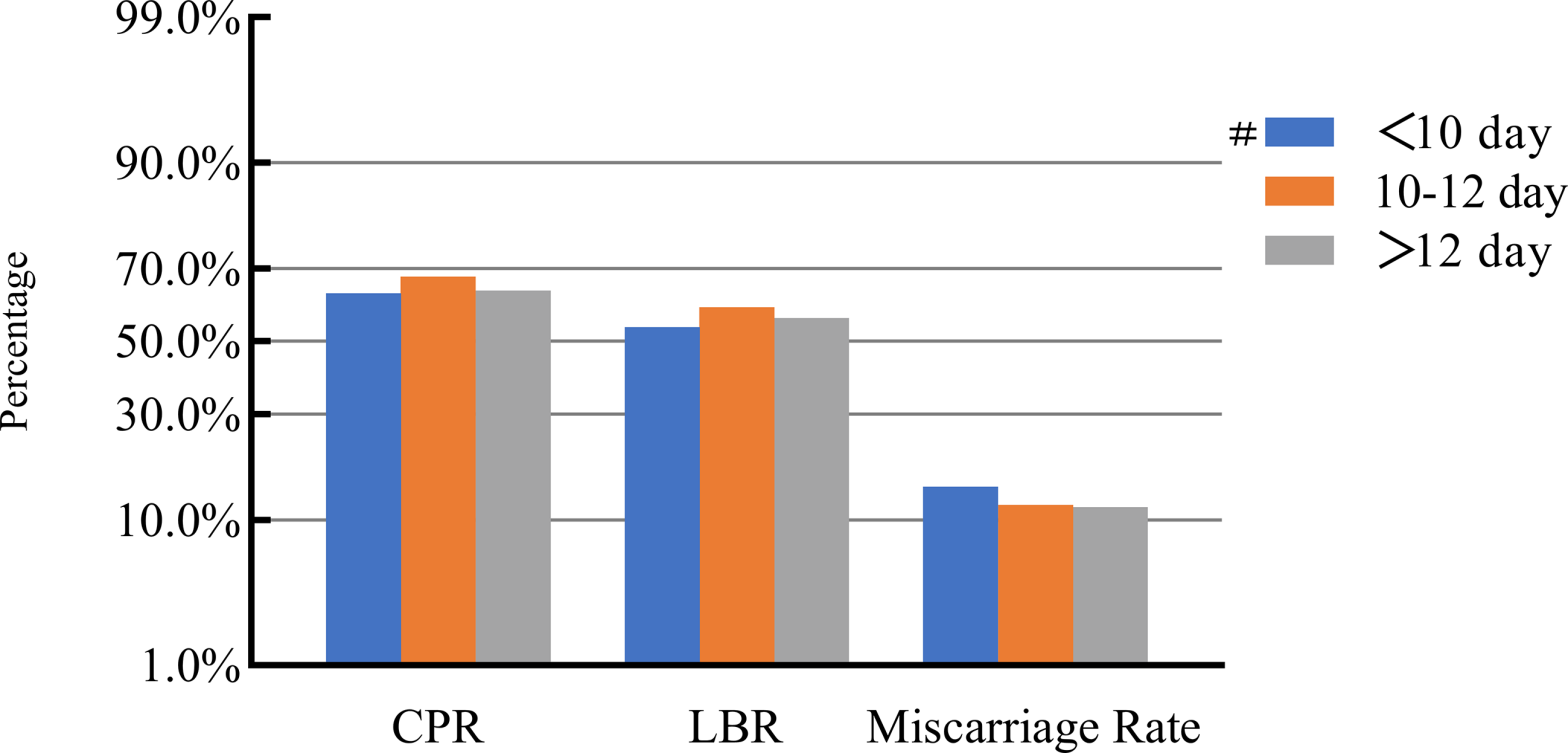
Association between stimulation duration and pregnancy outcomes. #, Reference group ^b^ *p*-values were calculated using a mixed logistic model adjusted for maternal age and BMI, method of endometrial preparation, endometrial thickness, transfer day of blastocyst and blastocyst quality. CPR, clinical pregnancy rate; LBR, live birth rate.

**Figure 4:**
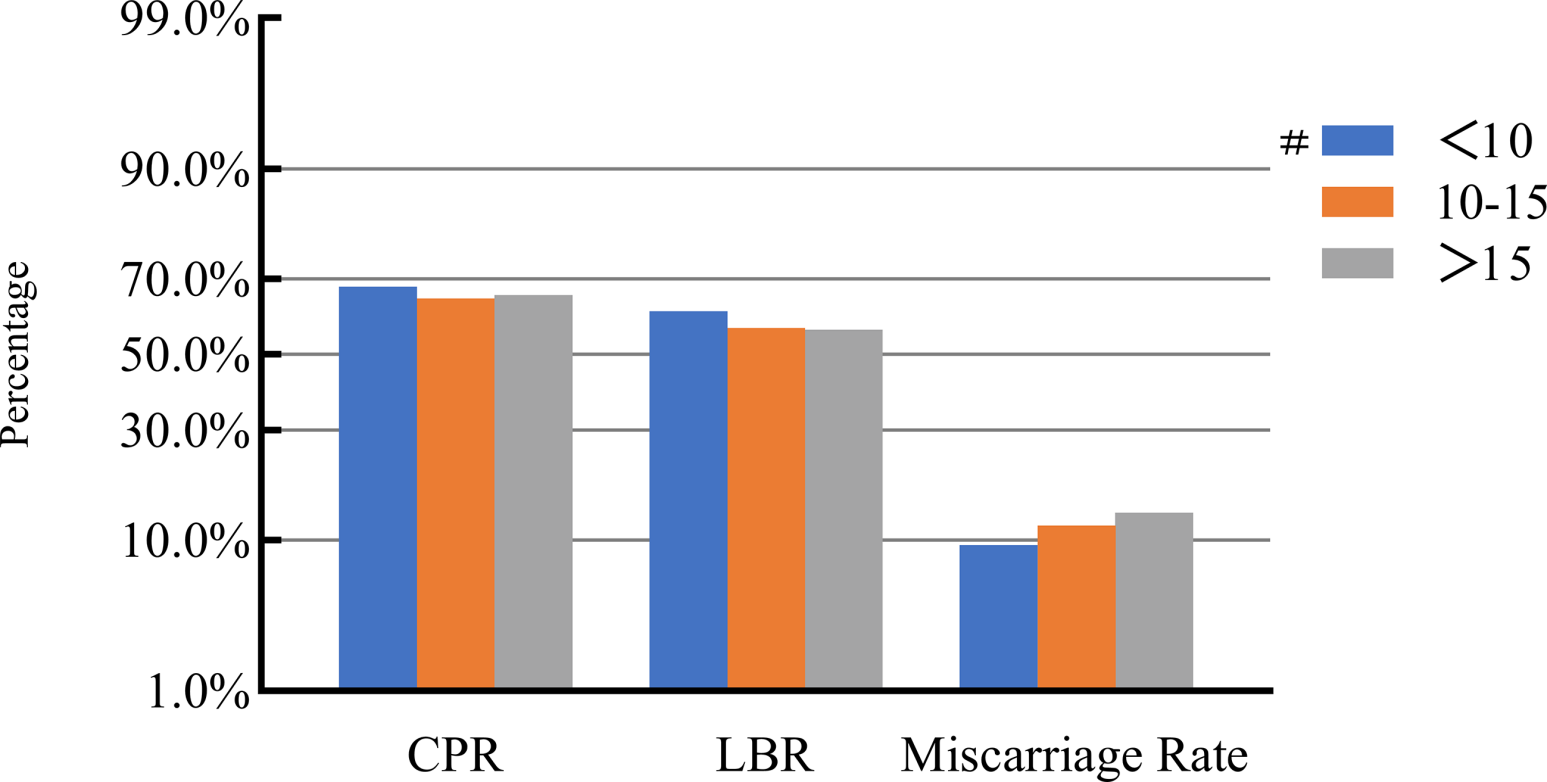
Association between the number of retrieved oocytes and pregnancy outcomes. #, Reference group ^b^ *p*-values were calculated using a mixed logistic model adjusted for maternal age and BMI, method of endometrial preparation, endometrial thickness, transfer day of blastocyst and blastocyst quality. CPR, clinical pregnancy rate; LBR, live birth rate.

**Figure 5:**
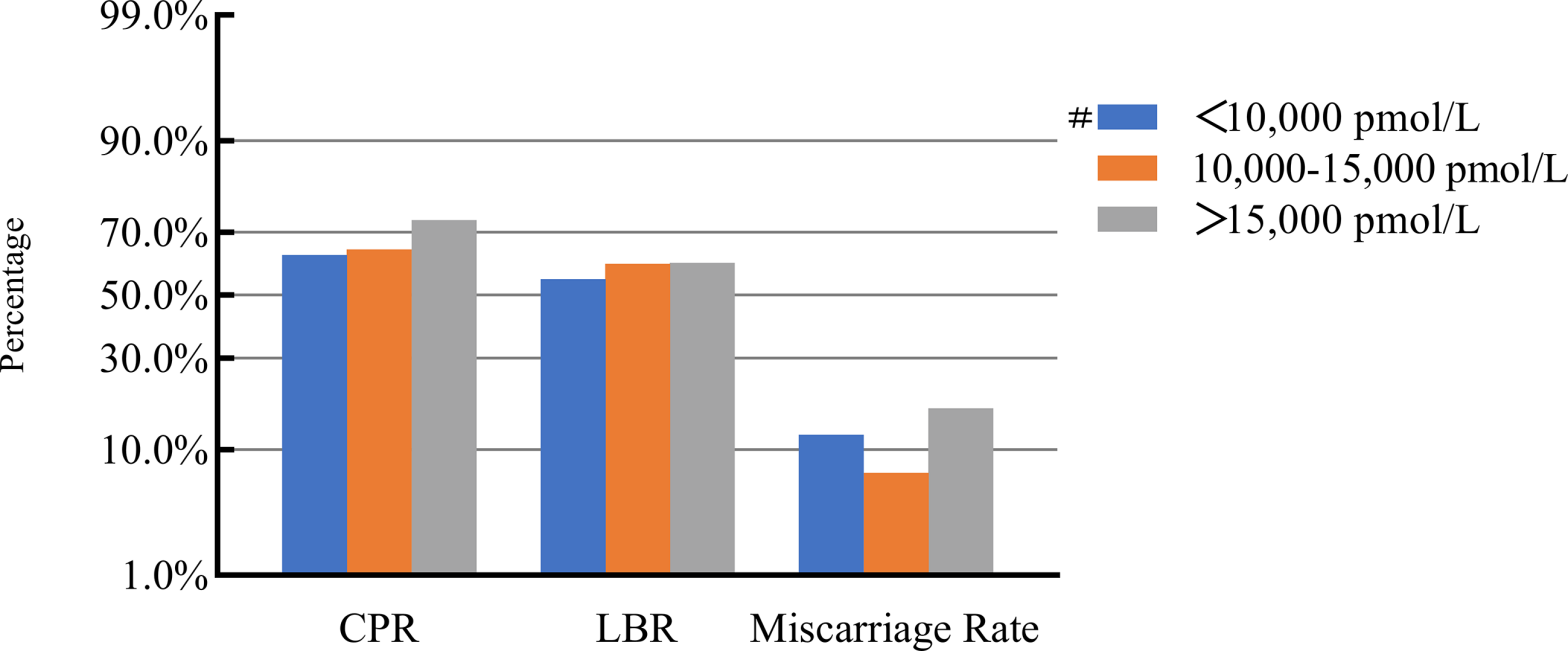
Association between maximal E_2_ levels and pregnancy outcomes. #, Reference group ^b^ *p*-values were calculated using a mixed logistic model adjusted for maternal age and BMI, method of endometrial preparation, endometrial thickness, transfer day of blastocyst and blastocyst quality. CPR, clinical pregnancy rate; LBR, live birth rate; E2, estradiol.

After adjusting for confounding factors, we found that COH parameters were not associated with clinical pregnancy outcomes (**Figures 1**–**5**). Clinical pregnancy rates ranged from 60.54% to 73.53% (^b^ *p* = 0.293) for the different stimulation methods, from 63.79% to 69.66% (^b^ *p* = 0.677) for different Gn dosages, from 63.53% to 67.86% (^b^ *p* = 0.827) for different stimulation durations, from 65.04% to 68.09% (^b^ *p* = 0.816) for different numbers of retrieved oocytes and from 63.01% to 73.40% (^b^ *p* = 0.432) for different maximal E_2_ concentrations. Corresponding live birth rates (LBRs) were 52.38%–64.71% (^b^ *p* = 0.320), 56.52%–58.43% (^b^ *p* = 0.969), 54.12%–59.69% (^b^ *p* = 0.605), 56.87%–61.70% (^b^ *p* = 0.600) and 55.25%–60.64% (^b^ *p* = 0.959), respectively. Similarly, no significant linear trends of relationship were seen between any of these factors and the rates of miscarriage (12.00%–13.48%, ^b^ *p* = 0.780; 9.01%–16.13%, ^b^ *p* = 0.358; 11.69%–14.82%, ^b^ *p* = 0.393; 9.38%–13.67%, ^b^ *p* = 0.498; and 7.02%–17.39%, ^b^ *p* = 0.089, respectively). The original data can be found in **Supplementary Tables 1, 2**.

## Discussion

The study comprehensively evaluated the incidence of *de novo* chromosomal abnormalities in blastocysts and clinical pregnancy outcomes for different COH parameters across 477 PGT cycles. The incidence of *de novo* chromosomal abnormalities was 22.98% in PGT-SR cycles and 22.63% in PGT-M cycles. Such abnormalities were found to increase with maternal or paternal age. It was found that the stimulation protocol, Gn dosage, stimulation duration, number of oocytes retrieved and peak E_2_ concentrations did not affect *de novo* aneuploidy rates or clinical pregnancy outcomes. We identified a significant age-dependent increase in *de novo* chromosomal abnormalities in the PGT-SR group. To our knowledge, this is the first study examining the relationships between COH parameters and *de novo* chromosomal abnormalities and clinical pregnancy outcomes following PGT.

In terms of ART, the high incidence of chromosomal abnormalities in the resultant embryos and the impact of such abnormalities on implantation have been widely discussed (18–20). Though PGT-A candidates have been studied frequently in previous studies, such individuals often harbor unexplained confounding factors that lead to high aneuploidy rates, thereby resulting in recurrent miscarriages and repeated implantation failures that necessitate PGT-A (21). Therefore, PGT-A samples were not suitable for accurately examining the relationship between ovarian stimulation and aneuploidy. Additionally, gametes produced by meiotic segregation in patients with unbalanced translocations potentially have an increased risk of aneuploidy. In addition to the direct effects of such translocations on the unbalanced chromosome, they may also affect the meiosis of other structurally normal chromosomes, leading to an increased risk of additional aneuploid gametes. This phenomenon is known as the inter chromosomal effect (ICE) (22). Many studies have attempted to confirm the ICE hypothesis, but it remains disputed (22–24). Therefore, we focused our study on *de novo* chromosomal abnormalities rather than pre-existing translocations or inversions. Additionally, patients undergoing PGT-M were chosen as a control group to verify whether ICE occurs.

It is well known that increased maternal age is an independent factor that negatively impacts the probability of obtaining a euploid embryo (2, 25). In line with this, we found that the incidence of *de novo* chromosomal abnormalities increased with maternal age. This might be because the risk of chromosomal abnormalities in oocytes and embryos increases with maternal age (26) largely due to recombination errors in early meiosis, defective spindle assembly checkpoints at MI and centromere cohesion loss (27). Similarly, our study found that older paternal age also has been associated with an increased incidence of *de novo* chromosomal abnormalities, which is consistent with previous studies. The association between aneuploidy rate and paternal age may be related to the level of sperm DNA fragmentation, which is theoretically believed that post-fertilization sperm DNA strand breaks can be repaired within the oocyte. However, the level of sperm DNA fragmentation deteriorates with age and the repair capacity of the embryo may not be able to compensate for the decrease in DNA quality, possibly leading to poorer embryo quality and aneuploidy rate increased (28, 29). In contrast, this study found no statistical differences between the PGT-SR and PGT-M groups in terms of the incidence of *de novo* chromosomal abnormalities across different age groups, suggesting that the ICE phenomenon does not occur. Therefore, despite finding evidence that increased *de novo* chromosomal abnormalities can occur in these conditions, they cannot be attributed to ICE.

Other than maternal or paternal age, chromosomal abnormalities have been thought to occur due to various aspects of the ART process, such as the ovarian stimulation protocols used for IVF. Undoubtedly, the goal of ovarian stimulation is to induce the ongoing development of multiple dominant follicles to obtain multiple mature oocytes (30, 31). Such ovarian stimulation is a critical aspect of IVF, particularly PGT cycles. In general, approximately 10–15 days of ovarian stimulation is required, during which E_2_ levels increase 10–20-fold compared with natural cycles. Some studies have proposed that oocytes obtained *via* natural or modified natural cycles are superior to those obtained *via* induced ovulation cycles (5). It is also believed that altered regulation of meiotic spindle alignment may occur due to the use of exogenous gonadotropins to stimulate the development of multiple follicles (32). Therefore, we examined whether varied COH parameters are correlated with an increase in *de novo* chromosomal abnormalities and worse pregnancy outcomes.

Our study demonstrated that the incidence of *de novo* chromosomal abnormalities and clinical pregnancy outcomes are not affected by the COH stimulation protocol used; a result consistent with previous findings (33). We found that the rate of *de novo* chromosomal abnormalities and miscarriages in those treated with a GnRH antagonist was slightly higher than in those treated with a GnRH agonist. However, this difference was not statistically different. Similarly, a Chinese birth cohort study of ART and birth defects reported that GnRH antagonist-based stimulatory regimens were associated with an increased risk of birth defects and that aneuploidy was a major factor resulting in these birth defects (34). Another study revealed that GnRH antagonist treatment was associated with higher aneuploidy rates in early aborted tissues and blastocysts than GnRH agonist treatment (35). These conflicting results may result from heterogenous study populations or variation in the specific methods used by different physicians in different IVF centers. Therefore, a strength of this study is that we focused on *de novo* chromosomal abnormalities rather than aneuploidy.

Previous theoretical studies have speculated that the administration of supraphysiological exogenous Gn interferes with natural follicle selection, potentially leading to chromosomal dysfunction and poor quality oocytes (36). It has also been proposed that low-dose Gn is associated with reduced aneuploidy rates in human preimplantation embryos (9, 37). In contrast, our findings indicate that the Gn dose, duration of ovarian stimulation treatment and peak of estrogen on hCG trigger day were not associated with the incidence of *de novo* chromosomal abnormality and clinical pregnancy outcomes. Other studies have demonstrated that different Gn dosages and the number of oocytes retrieved were not relevant to embryonic aneuploidy rates and pregnancy rates (38, 39). Moreover, another study reported that high-dose Gn leads to an increased risk of embryonic aneuploidy, but only in those with reduced ovarian reserve (6). Such reports of high-dose exogenous Gn leading to increased meiotic segregation errors in oocytes and increasing aneuploidy rates have not been confirmed. Other studies have reported that the duration of ovarian stimulation significantly affects LBRs of fresh cycles, potentially due to the adverse effects of high levels of Gn on endometrium receptivity - effects that are potentially reduced or eliminated in the case of frozen cycles (40). Therefore, in the absence of an alternative, milder method by which to stimulate ovarian maturation, conventional ovarian stimulation remains the recommended approach for most patients undergoing PGT. Our study reinforces previous findings that pregnancy rates are independent of the Gn dosage in frozen embryo transfer cycles.

In the study, we found that the duration of ovarian stimulation was not correlated with the incidence of *de novo* chromosomal abnormalities, while the > 12d group is lowest. It might because longer stimulation times are more conducive to the physiological process of cellular self-repair (41). This hypothesis has been corroborated in this study, as the incidence of *de novo* chromosomal abnormalities was reduced for the longer GnRH agonist protocol. Response to ovarian stimulation was assessed by the number of oocytes retrieved and maximal E_2_ concentrations on hCG trigger day. Supraphysiological levels of E_2_ are detected due to the maturation of multiple follicles, but more oocytes, and subsequently higher quality blastocysts, are required to obtain at least one euploid embryo for implantation. Clinicians have expressed concern that COH might affect the development and quality of oocytes, as multiple small, nondominant follicles will mature following exogenous Gn treatment (11). Labarta et al. reported (42) that a greater ovarian response, and thus number of oocytes retrieved, results in a greater number of resultant euploid embryos. Moreover, we found that the number of oocytes retrieved and maximal E_2_ concentrations on hCG trigger day were not correlated with *de novo* chromosomal abnormality rates and clinical pregnancy outcomes. These findings reinforce the lack of association between E_2_ concentrations and embryo quality or pregnancy outcomes seen in previous studies (43, 44). It had been suggested that a reduced oocyte yield represents an appropriate response to some forms of ovarian stimulation, as only the most competent follicles and oocytes can develop. However, older patients and those with a poor ovarian reserve should also be considered.

## Conclusion

In conclusion, this study revealed that total Gn dosage, ovarian stimulation duration, peak E_2_ concentrations and number of oocytes retrieved did not influence *de novo* chromosomal abnormality rates or clinical pregnancy outcomes. This study will provide clinicians with valuable information to consider when choosing an ovarian stimulation protocol. In addition to maternal age, AFC, baseline FSH levels and known causes of infertility, it is important to consider the potential to retrieve as many oocytes as possible to provide more embryos for biopsy. However, we do not advocate the use of high-dosage stimulation protocols, especially for patients at an increased risk of complications such as ovarian torsion and hyperstimulation syndrome. This is the first clinical study to examine the role of COH parameters in determining the incidence of *de novo* chromosomal abnormalities in Chinese IVF patients. Our findings may inform the development of novel strategies for ovarian stimulation during ART that promote both safety and efficacy. However, the mechanisms underlying *de novo* chromosomal abnormalities that occur during PGT cycles remain unclear.

## Limitations

This study was retrospective in nature, had a modest sample size and had a heterogenous study population. As such, larger prospective, multicenter and randomized controlled trials should be carried out to confirm our findings and further explore the mechanisms underlying the differential effects of different ovarian stimulation protocols.

## Data availability statement

The original contributions presented in the study are included in the article/supplementary files, further inquiries can be directed to the corresponding author.

## Ethics statement

The studies involving human participants were reviewed and approved by The review board of the Third Affiliated Hospital of Zhengzhou University. Written informed consent for participation was not required for this study in accordance with the national legislation and the institutional requirements.

## Author contributions

YL, RY and YG designed the study. PZ, JS and YZ were involved in the data extraction and analyses. YL and JS were involved in drafting this article. RP, JZ reviewed the data and article. All authors contributed to the article and approved the submitted version.
